# Historical Earthquakes and Their Socioeconomic Consequences in China: 1950–2017

**DOI:** 10.3390/ijerph15122728

**Published:** 2018-12-03

**Authors:** Xin He, Jidong Wu, Cailin Wang, Mengqi Ye

**Affiliations:** 1 Key Laboratory of Environmental Change and Natural Disaster, Ministry of Education, Beijing Normal University, No. 19, Xinjiekouwai Str., Beijing 100875, China; hxin2017@163.com (X.H.); wclzrdl@163.com (C.W.); yemengqi@mail.bnu.edu.cn (M.Y.); 2 Faculty of Geographical Science, Academy of Disaster Reduction and Emergency Management, Beijing Normal University, Beijing 100875, China

**Keywords:** earthquake disaster catalog, deaths, direct economic losses (DELs), spatiotemporal variation, earthquake magnitude, risk management

## Abstract

Understanding the spatiotemporal pattern of historical earthquake disasters and resultant socioeconomic consequences is essential for designing effective disaster risk reduction measures. Based on historical earthquake disaster records, this study compiles a Chinese earthquake disaster catalog (CH-CAT) that includes records of 722 earthquake disasters that occurred during 1950–2017 in the mainland of China. This catalog includes more complete data records than other existing global earthquake databases for China as a whole. Statistical results demonstrate that the number of earthquake disasters and the resultant direct economic losses (DELs) exhibit significant increasing trends (*p* < 0.01) over the studied 68-year period. Earthquake-induced deaths vary greatly between individual years and exhibit no significant trend. The Qinghai-Tibet seismic zone is the area with the highest frequency of earthquake disasters and the largest accumulated DELs, whereas the North China seismic zone is associated with the highest number of deaths. Among the 722 earthquake disasters, nearly 99.0% of deaths and 95.0% of DELs are attributable to 1.8% and 3.9% of the earthquake disasters, respectively. Approximately 54.2% of recorded earthquake disasters have earthquake magnitude (*Ms*) values between 5.0 and 5.9, while earthquake disasters with *Ms* greater than or equal to 7.0 account for 88.5% of DELs and 98.8% of deaths. On average, earthquake-induced DELs and deaths increase nonlinearly with increasing *Ms* per earthquake. DELs have a positive correlation with deaths and casualties on a logarithmic scale. This study further discusses that during different stages of socioeconomic development, changes in both exposure and vulnerability may be the major factors leading to change differences in earthquake-induced socioeconomic consequences. This study is a beneficial supplement to the global earthquake database and is useful for calibrating global or regional empirical loss models.

## 1. Introduction

China is located at the intersection of the Circum-Pacific and Mediterranean seismic belts. Within this earthquake-prone area, earthquakes in the mainland of China account for approximately one-quarter to one-third of the world’s earthquakes [1]. The distribution of earthquakes in China varies greatly; earthquake intensity can reach very high values in some cases and exhibits clear regional differences [2]. These characteristics often result in strong destructive forces that can cause severe damage. For example, the 1556 Huaxian earthquake with a magnitude (*Ms*) of 8.25 in Shaanxi was the deadliest earthquake on record; it killed approximately 0.8 million people [1]. The 1976 Tangshan earthquake caused 242,000 deaths [3]. The 2008 Wenchuan earthquake resulted in 69,227 deaths and 845 billion China Yuan (CNY) (US $124 billion) in direct economic losses (DELs) [4], amounting to 2.6% of China’s gross domestic product (GDP) for that year. The 2017 Jiuzhaigou earthquake caused 8.0 billion CNY of DELs, and Jiuzhaigou National Park was closed for seven months, seriously affecting tourism. Therefore, China is currently facing the challenge of reducing the number of deaths and the economic impacts caused by earthquakes. As a basis to understand the features of earthquake disasters, collating and analyzing the spatiotemporal characteristics of historical earthquake disasters and their socioeconomic impacts is essential for comprehensive disaster risk management.

There are several global and regional earthquake disaster databases that include earthquake-induced damage records for China, such as the Emergency Events Database (EM-DAT) (www.emdat.be) [5,6], Significant Earthquake Database (NGDC) (www.ngdc.noaa.gov) [7], NatCatSERVICE (www.munichre.com) [8], the PAGER-CAT—a composite earthquake catalog for calibrating loss models for the U.S. Geological Survey’s (USGS) Prompt Assessment of Global Earthquakes for Response (PAGER) system [9,10,11], and Catalog of Damaging Earthquakes in the World (Utsu) (www.iisee.kenken.go.jp). These earthquake disaster databases have provided a good foundation for analyzing global or national earthquake disaster characteristics [12,13,14,15,16,17,18,19]. However, it was found that global disaster databases inevitably omit a few disaster impact records, especially for early years [20,21,22]. Therefore, researchers have compiled historical earthquake catalogs that are as comprehensive as possible for further analysis [9,21,23,24,25]. Thereinto, some studies have compiled earthquake disaster databases for China at different time scales [26,27,28]. Unfortunately, these databases are not open to the public and have not been updated.

China has experienced frequent earthquakes since ancient times, and many researchers have studied earthquake disaster characteristics over the past several decades [12]. Dong et al. [29] noted that seismic activity is stronger in the west than in the east of China, whereas the earthquake disaster impacts exhibit the opposite pattern. Yuan et al. [26] reached the same conclusion and pointed out that the number of casualties has decreased over time, whereas the DELs have increased. Wang et al. [30] and Yang et al. [27] all noted that western China was the region with the highest frequency of earthquake disasters. These studies focused on the temporal and spatial distribution of earthquake disasters, and the analysis of earthquake disaster impacts was limited to relatively early years (before 2000). Therefore, with the update of earthquake disaster records, it is necessary to reanalyze the characteristics of earthquake disasters and their impacts based on a much longer time period, especially after the occurrence of a major earthquake such as the 2008 Wenchuan earthquake.

Thus, our aims are to map how historical earthquakes and their socioeconomic consequences have changed in space and time and to analyze the statistical characteristics of DELs and deaths. For this purpose, first, this study compiled a Chinese earthquake disaster catalog (CH-CAT) during 1950–2017 based on data from China Earthquake Administration (CEA). Then, we mapped the trends and distributions of historical earthquake disasters, DELs, and deaths. Lastly, we analyzed relationships among earthquake magnitude, DELs, and deaths. These results can help us to develop an empirical loss model and to design effective disaster reduction strategies across China.

## 2. Data Sources and Processing

### 2.1. Data Sources

Three types of data were used in this study, including seismic information data, earthquake-induced damage records, and socioeconomic statistic data (as shown in Table 1).

(1) Seismic information was acquired from the China Seismic Information database (www.csi.ac.cn), which is maintained by the CEA. It provides information about the date, time, latitude, longitude, *Ms*, depth of hypocenter, and location of earthquakes that have occurred in China since 780 B.C. The comprehensive seismic parameter catalog provides a reference list to avoid omitting earthquake disaster records.
(2) Records of earthquake-induced damage during the period 1950–2017 across 31 provinces in the mainland of China were collected from published books as well as online and relevant references provided by the CEA (Table 1). DELs caused by earthquakes (including ground motions, earthquake geological disasters, and earthquake secondary disasters) were measured by the damage of houses and other engineering measures, facilities, equipment, and items. Data on the DELs and numbers of deaths and injured people associated with earthquakes were obtained from following sources: (i) disaster data from 1950 to 1989 were obtained from A Comprehensive Compilation of Historical and Recent Earthquake Disaster Status in China [31]; (ii) Collection of Assessment Reports on Seismic Disaster Loss of Chinese Mainland provided detailed loss data from 1990 to 2010 [32,33,34,35]; (iii) records from 2011 to 2017 were from online annual seismic disaster loss reports provided by the CEA (www.cea.gov.cn). Earthquakes that occurred in Taiwan, Hong Kong, and Macao were excluded from this study due to a lack of statistical loss data.
(3) Socioeconomic statistic data (national GDP and consumer price index (CPI) data) were collected from the National Bureau of Statistics of China (www.stats.gov.cn).

### 2.2. Data processing

Frequency, DELs, and casualties are the three main characteristics considered when assessing disaster impacts [36]. These characteristics are used to represent the effects of earthquakes via the following method. (1) To analyze the spatiotemporal variations in DELs, the original reported DELs values were adjusted to the 2015 price level using the CPI. (2) The Mann-Kendall (M-K) trend test was used to detect trends in the earthquake disaster frequencies, DELs, and deaths [15]. In addition, spatial differences in the earthquake disaster impacts in five seismic zones in the mainland of China (i.e., North China, South China, Northeast China, Qinghai-Tibet, and Tianshan) were analyzed. 

## 3. Results

### 3.1. CH-CAT: Chinese Earthquake Disaster Catalog

CH-CAT includes seismic information, DELs, number of deaths, and number of injured for each earthquake disaster that occurred during 1950–2017 in the mainland of China. For this study, an earthquake disaster is defined as an earthquake that caused casualties and/or physical assets damage. Overall, the CH-CAT includes records of 722 independent earthquake disasters (i.e., one earthquake disaster is an independent record, which includes the main shock and its aftershock).

**Figure 1:**
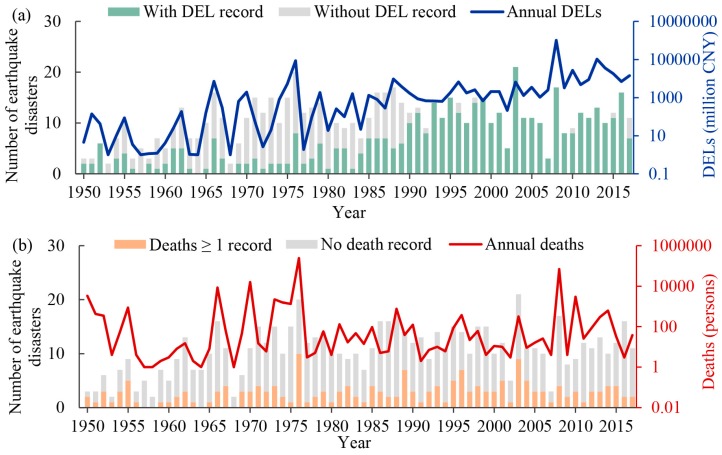
Annual variations in earthquake disaster numbers and earthquake-induced direct economic losses (DELs) (**a**) and deaths (**b**) over the period 1950–2017. Secondary y axes in the figure are on a logarithmic scale (note that a value of 1 has been added to the DELs and death values to avoid a value of zero).

Among the 722 earthquake disaster records, 443 (61.4%) earthquakes were associated with DEL reports, and the other 289 disasters caused at least some form(s) of casualties (bars in Figure 1a). Notably, since 1990, the Chinese government began to build a comprehensive disaster statistics system [31]. Consequently, during 1950 to 1989, only major earthquake disasters feature reported DELs. DELs for some earthquakes were not assessed because of the limited physical asset damage of the earthquake (corresponding to the ‘without DEL record’ bar in Figure 1a). Furthermore, 189 earthquakes (26.5% of the earthquake disasters) resulted in deaths (bar in Figure 1b). Finally, there were 152 earthquake disasters from which both deaths and DELs were recorded.

### 3.2. Spatiotemporal Characteristics of Earthquake Disaster Frequency 

**Figure 2:**
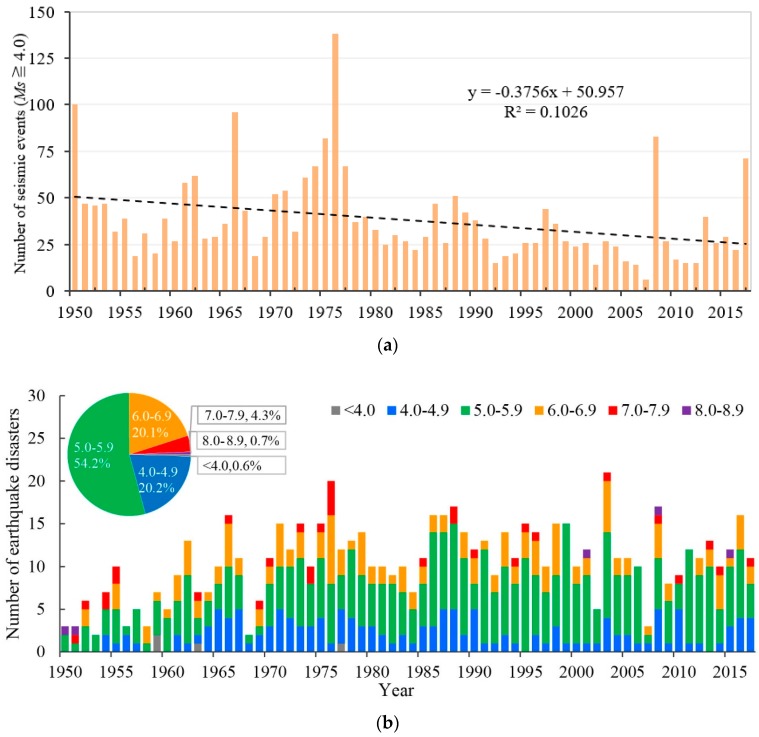
Number of seismic events that occurred in the mainland of China (**a**) and earthquake disaster records in Chinese earthquake disaster catalog (CH-CAT) during 1950–2017 by earthquake magnitude (bar) (**b**). The pie chart in **b** represents the proportion of earthquake disasters for each *Ms* interval. Seismic events records were from the China Earthquake Administration (CEA).

As seen in Figure 2 compared with a slightly decreasing trend in the number of seismic events that occurred in the mainland of China (Figure 2a), there is an increasing trend (*p* < 0.01, M-K trend test), but with fluctuations, in the annual total number of earthquake disasters in China during 1950–2017 (Figure 2b); for example, there is a decreasing trend (*p* < 0.10) during 1986–2017 (Figure 2b). Regarding the earthquake magnitude level, significant increasing trends are also observed for earthquakes with magnitudes of 5.0 ≤ *Ms* ≤ 5.9 (*p* < 0.01) and 6.0 ≤ *Ms* ≤ 6.9 (*p* < 0.10) during 1950–2017. Among the 722 earthquake disasters, 54.2% of them had magnitudes of 5.0 ≤ *Ms* ≤ 5.9, and earthquakes with magnitudes of 4.0 ≤ *Ms* ≤ 4.9 and 6.0 ≤ *Ms* ≤ 6.9 each accounted for approximately one-fifth of the recorded earthquake disasters. 

**Figure 3:**
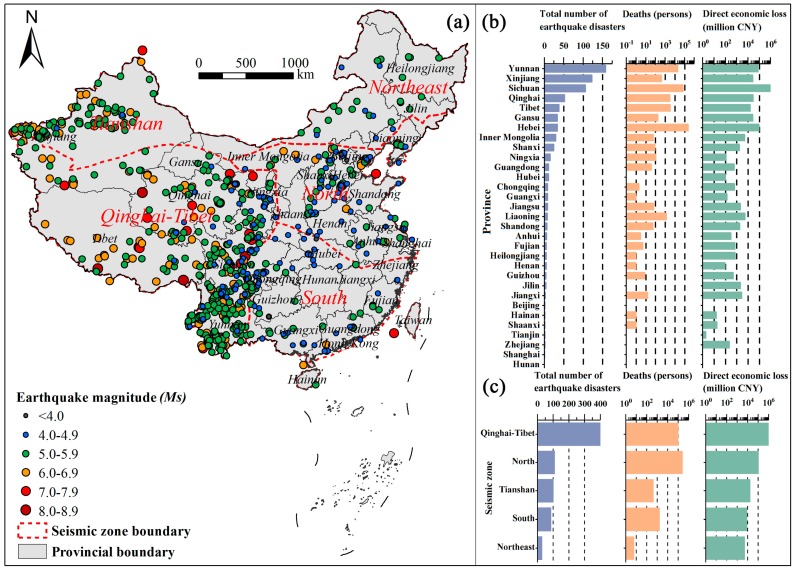
Spatial distribution of earthquake disasters (**a**) and their corresponding consequences by province (**b**) and by seismic zone (**c**) in the mainland of China during 1950–2017. CNY is China Yuan.

**Figure 4:**
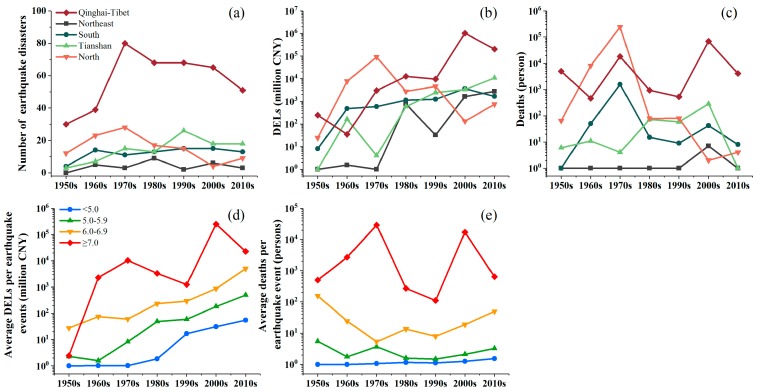
Decadal variability in the number of earthquake disaster, deaths, and DELs by seismic zone (**a–c**) and decadal average DELs and deaths per earthquake event by earthquake magnitude (**d**,**e**). The y axes in (**b**–**e**) are on the logarithmic scale, and a value of 1 was added to the DELs and deaths values to avoid a value of zero. Legends for **b** and **c** are the same as that for **a**, and the legend for **e** is the same as that for **d**.

The distribution of earthquake disasters in the mainland of China exhibits multiscale spatial characteristics (Figure 3). At the seismic zone level, earthquake disasters are widely distributed in the Qinghai-Tibet seismic zone, which accounted for 55.5% of all earthquake disasters, followed by the Tianshan (15.0%) and North China (14.9%) seismic zones. Decadal frequency statistics show that the accumulated number of earthquake disasters in the Tianshan seismic zone exhibits an increasing trend (*p* < 0.05), while similar increasing trends in the Qinghai-Tibet (*p* = 0.88), Northeast China (*p* = 0.65), and South China (*p* = 0.22) seismic zones were not significant. Furthermore, the North China seismic zone exhibits a weak downward trend (*p* = 0.23). (Figure 4a). 

At the province level, earthquake disasters affected almost all provinces in China during the period 1950–2017. The number of earthquake disasters in the provinces of Yunnan (158), Xinjiang (123), and Sichuan (106) accounted for 53.6% of the total number of earthquake disasters. Additionally, nearly half of the provinces experienced more than ten earthquake disasters. Earthquakes with *Ms* equal to or greater than 8.0 occurred in Xinjiang (one event), Sichuan (one event), and Tibet (three events). Although Yunnan Province experienced the most earthquake disasters, the maximum *Ms* was 7.8 during 1950–2017.

### 3.3. Change Trends and Distribution of Earthquake-Induced DELs and Deaths

**Figure 5:**
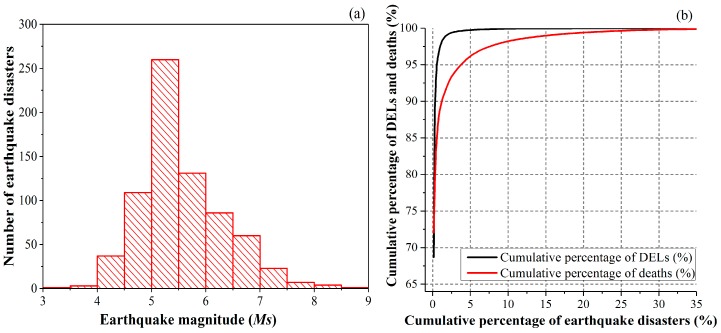
Frequency distribution of *Ms* (**a**) and cumulative distribution of earthquake-induced DELs and deaths (**b**).

The magnitude distribution of the 722 earthquake disasters is close to a normal distribution (Figure 5a). In total, the recorded earthquakes resulted in approximately 1.393 trillion CNY (223.7 billion US$ in 2015, 1 US$ = 6.2274 CNY) of DELs and 352,282 deaths during 1950–2017 in the mainland of China. In detail, 99.0% of the deaths were associated with 1.8% of the earthquake disasters, and 95.0% of the DELs were associated with 3.9% of the earthquake disasters (Figure 5b).

The 10 most severe earthquake disasters in the past 68 years, ranked based on their DELs and deaths, are listed in Table 2. These earthquake disasters caused the greatest DELs and the most deaths. Among all the earthquake disasters, the 10 costliest earthquake disasters accounted for 90.6% of the total DELs, and the 10 deadliest earthquake disasters accounted for 98.5% of the total deaths. The 2008 *Ms* 8.0 Wenchuan earthquake caused the greatest DELs (i.e., 1.003 trillion CNY. The 1976 *Ms* 7.8 Tangshan earthquake, which caused 242 thousand deaths, was the deadliest earthquake in the past 68 years. Furthermore, approximately 72.0% of the total DELs were caused by the 2008 Wenchuan earthquake, and 68.7% of the total deaths were caused by the 1976 Tangshan earthquake.

For the DEL trend, as shown in Figure 1a, the DELs clearly exhibit an increasing trend over the past 68 years (*p* < 0.01). Additionally, the DELs also show a significant increasing trend during 1990–2017 (*p* < 0.01). The frequency of earthquake disasters with severe DELs has increased since 2008. For example, there was only one year in which the DELs were greater than 20 billion CNY before 2008, while there were five years in which the DELs were greater than 20 billion CNY after 2008. The total DELs in the period 2008–2017 were approximately 7.8 times higher than those in the period 1950–2007. By earthquake magnitude, the decadal average DELs per earthquake event with magnitudes of *Ms* < 5.0, 5.0 ≤ *Ms* ≤ 5.9 and 6.0 ≤ *Ms* ≤ 6.9 showed significant upward trends (*p* < 0.01); a similar trend was observed for *Ms* ≥ 7.0 but was not statistically significant (*p* = 0.13) (Figure 4d).

The total number of deaths due to earthquake disasters varied greatly between individual years and were often concentrated around large-scale events (Figure 1b). More deaths occurred over the period 1950–1976 than during the period 1977–2017. There were three years with over 5000 deaths during the period 1950–1976. In comparison, in the 40 years after 1976, there was only one year with over 5000 deaths. Decadal average deaths per earthquake event also varied among the earthquake magnitude intervals and exhibited no significant trends (Figure 4e).

Changes in DELs and deaths feature large regional differences at both the seismic zone scale and the provincial scale. At the seismic zone scale, the Qinghai-Tibet seismic zone accounts for 90.0% of the earthquake-related DELs, and the North seismic zone accounts for 71.5% of earthquake disaster deaths (Figure 3c). The decadal accumulated DEL statistics show that the total DELs of earthquake disasters in the Tianshan, Qinghai-Tibet, Northeast, and South China seismic zones show increasing trends (*p* < 0.05) (Figure 4b). In contrast, there are no significant trends for decadal accumulated deaths in any seismic zone (Figure 4c). At the provincial level, over the past 68 years, Sichuan Province has experienced the highest DELs (amounting to 1.095 trillion CNY, i.e., 175.8 billion US$), followed by Hebei Province (93.5 billion CNY, i.e., 15.0 billion US$) and Yunnan Province (79.1 billion CNY, i.e., 12.7 billion US$). The most deaths occurred in Hebei Province (at least 250,123 deaths), followed by Sichuan Province (72,336 deaths) and Yunnan Province (20,920 deaths) (Figure 3b). 

### 3.4. Statistical Relationship between Earthquake-Induced Deaths, DELs, and Ms

**Figure 6:**
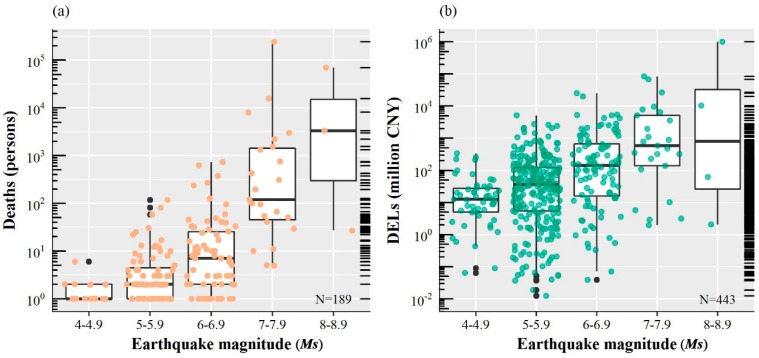
The relationship between *Ms* and earthquake-induced DELs (**a**) and deaths (**b**). The earthquake disasters plotted in the figures are associated with at least one death (**a**) or with reported DELs (**b**).

Earthquake-induced DELs and deaths increase steadily with increasing earthquake magnitude, on the denary logarithmic scale (Figure 6). The median numbers of deaths (in absolute values) for the earthquake magnitude intervals of 4.0 ≤ *Ms* ≤ 4.9, 5.0 ≤ *Ms* ≤ 5.9, 6.0 ≤ *Ms* ≤ 6.9, 7.0 ≤ *Ms* ≤ 7.9, and 8.0 ≤ *Ms* ≤ 8.9 are 1.0, 2.0, 7.0, 108.5, and 3300.0 persons, respectively. Similarly, the corresponding median numbers of DELs (in absolute values) are 12.9, 36.3, 154.8, 678.7, and 5182.0 million CNY, respectively. The median number of DELs for 7.0 ≤ *Ms* ≤ 7.9 is approximately 18.7 times higher than that for 5.0 ≤ *Ms* ≤ 5.9. In addition, earthquake disasters with magnitudes of 5.0 ≤ *Ms* ≤ 5.9 caused only 3.4% of the accumulated DELs and 0.2% of the accumulated deaths during 1950–2017. In contrast, 88.5% of DELs and 98.8% of deaths were caused by earthquakes with magnitudes greater than or equal to 7.0.

Most earthquakes are associated with relatively low losses, and only a few earthquakes cause enormous losses. Among the 189 earthquake disasters that caused death, half of them resulted in less than four deaths, and only 4.8% of earthquake disasters resulted in more than one thousand deaths (Figure 6a). Most earthquake disasters resulted in DELs between one million to one billion CNY (Figure 6b).

**Figure 7:**
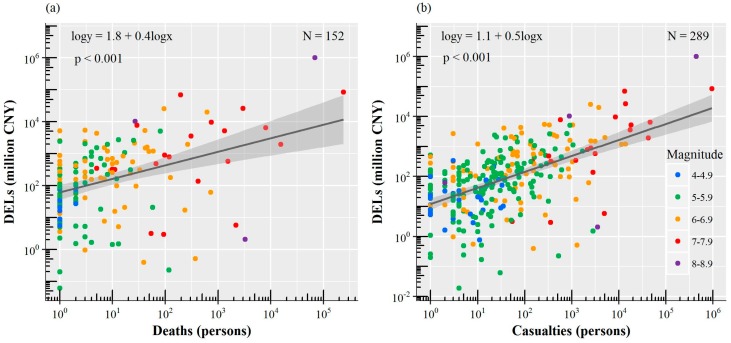
Earthquake-induced DELs versus deaths (**a**) and casualties (**b**) by earthquake magnitude. (**a**) The earthquake disasters plotted in these figures are earthquakes with both at least one death and reported DELs. (**b**) Same as (**a**), but for casualties. Nonparametric smooth curves with 95% confidence interval are shown in gray.

On the logarithmic scale, the results show a positive correlation between earthquake-induced DELs and deaths/casualties, especially for casualty-DELs relationship (Figure 7). Earthquake-induced DELs increase with increasing deaths and casualties. However, the number of deaths varies greatly and does not always correspond well to DELs due to the built environment, wealth, or other factors. For example, the 8.6 *Ms* 1950 Chayu earthquake caused 3300 deaths and ranks fifth on the list of the 10 deadliest earthquake disasters (Table 2), but it does not rank among the 10 earthquakes with the largest DELs. 

Overall, earthquake magnitude is an important factor for determining the earthquake-induced damage and the loss–*Ms* relationship presents a nonlinear function. Moreover, there is a close relationship between casualties and DELs for earthquakes. 

## 4. Discussion

### 4.1. Improvement of CH-CAT compared with Other Global Earthquake Disaster Datasets

**Figure 8:**
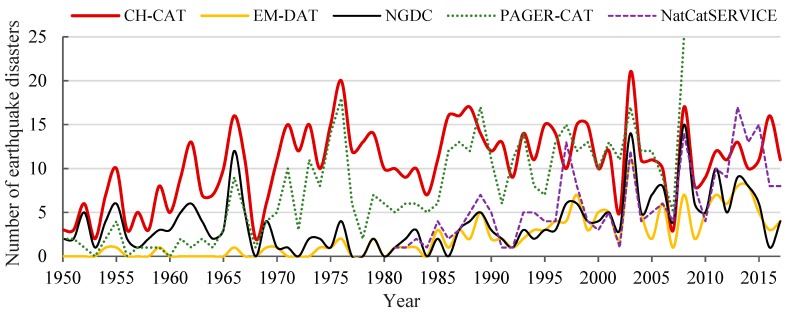
Annual variability in the number of earthquake disaster records in CH-CAT compared with other global earthquake disaster datasets (i.e., Significant Earthquake Database (NGDC), Emergency Events Database (EM-DAT), PAGER-CAT—a composite earthquake catalog for calibrating loss models for the U.S. Geological Survey’s (USGS) Prompt Assessment of Global Earthquakes for Response (PAGER) system, and NatCatSERVICE) during 1950–2017.

For China’s mainland, Figure 8 shows that the annual total number of earthquake disaster records in CH-CAT is mostly higher than those of the other four existing global earthquake disaster databases (i.e., NGDC, EM-DAT, PAGER-CAT, and NatCatSERVICE). The differences among these datasets are as follows. (1) CH-CAT has more extensive earthquake disaster impact records than the other databases, especially in the early periods (before 1990). (2) The numbers of records in NGDC and EM-DAT are generally lower than those in the other datasets. (3) PAGER-CAT has a relatively high number of records because PAGER-CAT incorporates eight global earthquake catalogs, but the available data extend only until 2008 and need further updating. (4) NatCatSERVICE provides access to data on earthquake disasters since 1980, and the records are more comprehensive in recent years. Evidently, over the period 1950–2017, NGDC and EM-DAT have detailed records of major earthquake disasters in the mainland of China but substantially underreport earthquake disasters associated with smaller losses (Table 3). However, small-loss earthquake records represent an important source of information for understanding the contributions of historical DELs and deaths. Therefore, the development of CH-CAT involved the collection of earthquake disaster records that was as comprehensive as possible.

In addition to missing records, differences in the criteria used in these databases are another important reason for differences among the datasets. For example, for a disaster to be entered into EM-DAT, it must fulfill at least one of the following criteria: (1) ten or more people were reportedly killed; (2) one hundred (100) or more people were reportedly affected; (3) a state of emergency was declared; or (4) a call for international assistance was issued (www.emdat.be). Furthermore, the criteria for defining an earthquake disaster event also vary. For example, in the case of the 2008 Wenchuan earthquake and its aftershocks, we found that there were eight damage records in PAGER-CAT but only one record in CH-CAT. This discrepancy may be the reason why the total number of earthquake disasters in PAGER-CAT is higher than that in CH-CAT in certain years (i.e., the years 2008 and 1997).

Overall, compared with other global earthquake disaster catalogs, CH-CAT is a more complete earthquake disaster catalog for the mainland of China and provides a sound basis for analysis of the temporal and spatial distribution of earthquake disasters and their consequences in the mainland of China during 1950–2017.

### 4.2. Effects of Major Earthquakes on Disaster Impact Trends

Extremely strong earthquakes have destructive consequences for society. The top 10 earthquake disasters accounted for more than 90.0% of the accumulated DELs and deaths during the past 68 years and had severe impacts on the temporal and spatial distribution of DELs and deaths. It is remarkable that an occasional large-impact earthquake could drastically change the trend of the disaster impact and focal points of studies. 

Table 4 summarizes and compares the results of decadal average annual DELs and deaths with and without some destructive earthquakes. First, there are no consistent trends in the death and DEL data over the past several decades in terms of actual total damage. For example, the annual average number of deaths in the 1970s was 26,313; it then decreased to only 68 in the 1990s but reached 6968 in the 2000s. This fluctuation is explained by the occurrence of large earthquake disasters, and the peak values correspond to years when large earthquake disasters occurred, such as the 1966 Ningjin earthquake, the 1976 Tangshan earthquake, the 2008 Wenchuan earthquake, and the 2010 Yushu earthquake. Therefore, we recalculated the annual average losses and excluded earthquake disasters that caused more than 500 deaths or DELs ranking within the top 10 overall losses (as listed in adjusted values in Table 4). After this adjustment, there was an increasing trend of average annual DELs (*p* < 0.05) and a decreasing trend of average annual deaths (*p* < 0.13), but the latter did not pass the significance test. 

### 4.3. Relationship between Economic Development and Earthquake Disaster Impacts

An earthquake disaster is closely related to hazards, exposure, and vulnerability, and its severity depends on natural features and on socioeconomic development. The consequences of an earthquake disaster can change due to changes in exposure and vulnerability during different stages of economic development [37]. In earlier times, the population and economy were less exposed to natural hazard-induced disasters, but the population was more vulnerable to the effects of earthquake disasters due to poorly built environments. For example, 70.0% of the 10 deadliest earthquake disasters occurred before 1980 (Table 2). 

Currently, rapid economic development as well as population growth corresponds to increased exposure to earthquakes. The total GDP located in the most seismically hazardous areas reached 6.2 trillion CNY in the year 2010, with an average annual rate of increase of 11.3% over two decades [38]. The population of the most seismically hazardous areas increased by 32.5 million, at a significant rate of 33.6% [39]. Increased exposure causes greater seismic risk in the mainland of China in that it causes more earthquakes to become disasters. DELs due to earthquake disasters have increased in the past several decades, as demonstrated above.

However, the vulnerability of residents and the economy has decreased due to growing investments in risk reduction [22]. With increased economic development, fewer disaster deaths have occurred in China [40]. An increase in income has been an important factor in reducing deaths because it has increased the private demand for safety [22]. Higher incomes allow people to employ individual additional precautionary measures to mitigate risks around them [41,42,43] and are also responsible for many engineering improvements. Overall, socioeconomic development increases wealth exposure to earthquakes, which is an important reason for the increase in DELs. This pattern suggests that earthquake DELs will increase with economic growth. However, we argue that increased efforts should be expended in order to further explore and deepen our understanding of how earthquake disaster damage varies with earthquake magnitude, asset value exposure [44], and vulnerability as a function of physical, social, and economic factors. 

### 4.4. Policy Implications

Earthquake disasters are important natural hazard-induced disasters in China. Along with the recent socioeconomic development and population growth, both the exposure and the vulnerability of disaster-bearing bodies have changed dramatically. Earthquake disasters and their impacts on human society have attracted the attention of the international community. However, because our ability to predict earthquakes is currently limited due to the large uncertainties associated with earthquake forecasts, the government is under great pressure. Coupling earthquake forecasting/prediction with disaster-reduction measures may become a future priority [37]. In this situation, the results obtained by studying past earthquakes may provide us with the confidence to provide effective recommendations in the future. 

It is important to customize disaster-reduction measures based on geographic differences and the different characteristics of earthquake disaster areas. For the regions regarded as seismically inactive and those that are frequently affected by earthquakes but have experienced little financial loss, more attention should be paid to investing in buildings to reduce the vulnerabilities of properties and residents. The Qinghai-Tibet, Tianshan, and North China seismic zones suffer from frequent earthquake disasters. Sichuan and Hebei Provinces represent the high-cost areas in this region and are places where large earthquake disasters have occurred. These factors usually correspond to greater exposure. Therefore, in addition to implementing engineering measures, it is important to reduce risk by educating and training the public (i.e., earthquake emergency drills), making earthquake insurance available, and implementing other measures. The Chinese government has been developing earthquake emergency rescue capabilities since the start of the 21st century. The Chinese rescue team has played important roles in rescue and relief actions at home and abroad [37]. After the 2008 Wenchuan earthquake, the government developed and improved a series of mechanisms and systems to prepare for catastrophes [44,45,46], and the relevant departments have enhanced their ability to monitor and mitigate disasters. The effectiveness of such emergency management was reflected in the aftermath of the 2013 Lushan earthquake. 

## 5. Conclusions

This study compiled an earthquake disaster catalog for China (CH-CAT) that includes 722 earthquake-induced disaster events from 1950–2017; CH-CAT is a more complete earthquake disaster catalog than the existing global datasets. Based on the data from CH-CAT, the statistical socioeconomic consequences are as follows:

(1) Earthquake disasters caused approximately 1.393 trillion CNY (223.7 billion US$ in 2015) in DELs and 352,282 deaths during 1950–2017 in China. Among the 722 earthquake disasters, half of the earthquake disasters had *Ms* of 5.0 ≤ *Ms* ≤ 5.9. However, earthquake-induced damage was mostly produced by large earthquakes. The 10 costliest earthquake disasters accounted for 90.6% of the total DELs, and the 10 deadliest earthquake disasters accounted for 98.5% of the total deaths from all earthquake disasters. By province, Yunnan Province experienced the highest number of earthquake disasters, Sichuan Province experienced the greatest DELs, and Hebei Province experienced the most deaths.
(2) The number and DELs of earthquake disasters exhibited increasing trends with time (*p* < 0.01) but were subject to disaster impacts reporting quality over time. By earthquake magnitude, the decadal average DELs per event show t increasing trends with time (*p* < 0.01) for *Ms* < 5.0, 5.0 ≤ *Ms* ≤ 5.9, and 6.0 ≤ *Ms* ≤ 6.9. By seismic zone, the Qinghai-Tibet seismic zone was the area with the highest frequency of earthquakes and the largest DELs, and the North China seismic zone was the area with the highest number of deaths. The decadal accumulated DELs of the earthquake disasters in Tianshan, Qinghai-Tibet, Northeast, and South China seismic zone showed increasing trends with time (*p* < 0.05).
(3) Statistically, earthquake-induced DELs and deaths increased nonlinearly with increasing *Ms* per earthquake. Most earthquake were associated with relatively low losses, whereas a few large earthquakes caused enormous losses. Among the 722 earthquake disasters in CH-CAT, only 26.5% resulted in deaths, and among these earthquakes, half resulted in fewer than four deaths. Furthermore, most earthquake disasters resulted in DELs between one million to one billion CNY. Moreover, on the logarithmic scale, earthquake-induced DELs had a positive correlation with deaths and casualties.

Overall, changes in earthquake disaster frequency and resultant DELs suggest that earthquakes will exert greater impacts on society, especially when facing increasing exposure. The spatiotemporal characteristics of earthquake-induced socioeconomic impacts discussed above provide a good foundation for customizing disaster-reduction measures based on geographic differences, while the earthquake-induced damage and its relationship with hazard also provide a basis for calibrating loss estimation models. 

## Figures and Tables

**Table 1:** Main data sources used in this study.

Data	Source	Author	Type	Time Period	Characteristics and Use
Seismic information	China Seismic Information (www.csi.ac.cn)	China Earthquake Administration (CEA)	Online	1950–2017	Basic seismic information for China
Earthquake-induced damage records	A Comprehensive Compilation of Historical and Recent Earthquake Disaster Status in China	Lou [31]	Book	1950–1989	Disaster records include maximum intensity, number of deaths, number of injured, and direct economic losses (DELs)
	Collection of Assessment Reports on Seismic Disaster Loss of Chinese Mainland	CEA [32,33,34,35]	Book	1990–2010	
	Chinese annual seismic disaster loss reports (www.cea.gov.cn)	CEA	Online	2011–2017	
Socioeconomic statistic data	Consumer price index (CPI) (www.stats.gov.cn)	National Bureau of Statistics of China	Online	1950–2017	DELs are converted to the 2015 price level

**Table 2:** The 10 most severe earthquake disasters, ranked based on their DELs and deaths, respectively, during 1950–2017.

Year	Location	*Ms*	DELs (Billion CNY)	Year	Location	*Ms*	Deaths (Persons)
2008	Wenchuan, Sichuan	8.0	1003.4	1976	Tangshan, Hebei	7.8	242,000
1976	Tangshan, Hebei	7.8	84.6	2008	Wenchuan, Sichuan	8.0	69,227
2013	Lushan, Sichuan	7.0	68.8	1970	Tonghai, Yunnan	7.8	15,621
2010	Yushu, Qinghai	7.3	26.2	1966	Ningjin, Hebei	7.2	8064
2013	Minxian, Gansu	6.7	25.3	1950	Chayu, Tibet	8.6	3300
2014	Ludian, Yunnan	6.5	20.1	2010	Yushu, Qinghai	7.3	2968
2015	Tibet (Nepal earthquake)	8.1	10.3	1973	Luhuo, Sichuan	7.6	2199
1988	Lancang, Yunnan	7.6	9.5	1974	Daguan, Yunnan	7.1	1541
2017	Jiuzhaigou, Sichuan	7.0	7.8	1975	Haicheng, Liaoning	7.3	1328
1966	Ningjin, Hebei	7.2	6.5	1988	Lancang, Yunnan	7.6	748

**Table 3:** Comparison of the total number and time scale of earthquake disasters in five databases for the mainland of China.

Name	Number of Earthquake Disaster Records	Number of Earthquakes with DEL Records	Time Period
CH-CAT	722	443	1950–2017
EM-DAT	152	66	1950–2017
NGDC	257	36	1950–2017
PAGER-CAT	438	-	1950–2008
NatCatSERVICE	222	-	1980–2017

Notes: DELs were adjusted to the 2015 price level. CH-CAT: Chinese earthquake disaster catalog; NGDC: Significant Earthquake Database; EM-DAT: Emergency Events Database; PAGER-CAT: a composite earthquake catalog for calibrating loss models for the U.S. Geological Survey’s (USGS) Prompt Assessment of Global Earthquakes for Response (PAGER) system.

**Table 4:** Earthquake disaster impacts (DELs and deaths) by decade, 1950–2017.

Time Period	Average Annual DELs (Billion CNY)		Average Annual Deaths (Persons)	
	Actual Value	Adjusted Value	Actual Value	Adjusted Value
1950–1959	0.0	0.0	502	99
1960–1969	1.1	0.2	861	55
1970–1979	9.6	1.1	26,313	44
1980–1989	1.8	0.8	110	35
1990–1999	1.8	1.8	68	68
2000–2009	96.5	3.1	6968	45
2010–2017	27.4	7.5	510	12

Note: Actual value means average annual earthquake disaster impact (i.e., DELs or deaths) calculated based on the 722 events. Adjusted value for DELs represents values excluding earthquake disasters that ranked within the top 10 DELs. Adjusted value for deaths represents values excluding earthquake disasters that caused more than 500 deaths.
